# Supplementary material for: Functional Annotation Genome Unravels Potential Probiotic Bacillus velezensis Strain KMU01 from Traditional Korean Fermented Kimchi
Source: Foods. 2021 Mar 9;10(3):563. doi: 10.3390/foods10030563 (PMC7998376; doi:10.3390/foods10030563)
Supplement: Supplementary file 1 [file foods-10-00563-s001.pdf]

Fig. S1. Phylogenetic trees based on the entire gene sequences of adk, ccpA, glpF, gmk, ilvD, pur, spoOA, and tpi showing the phylogenetic relationships between strain KMU01 and other *Bacillus*. The phylogenetic tree was constructed with the maximum likelihood method. Branches with bootstrap values 50 % were collapsed. The distance scales are shown under the trees.

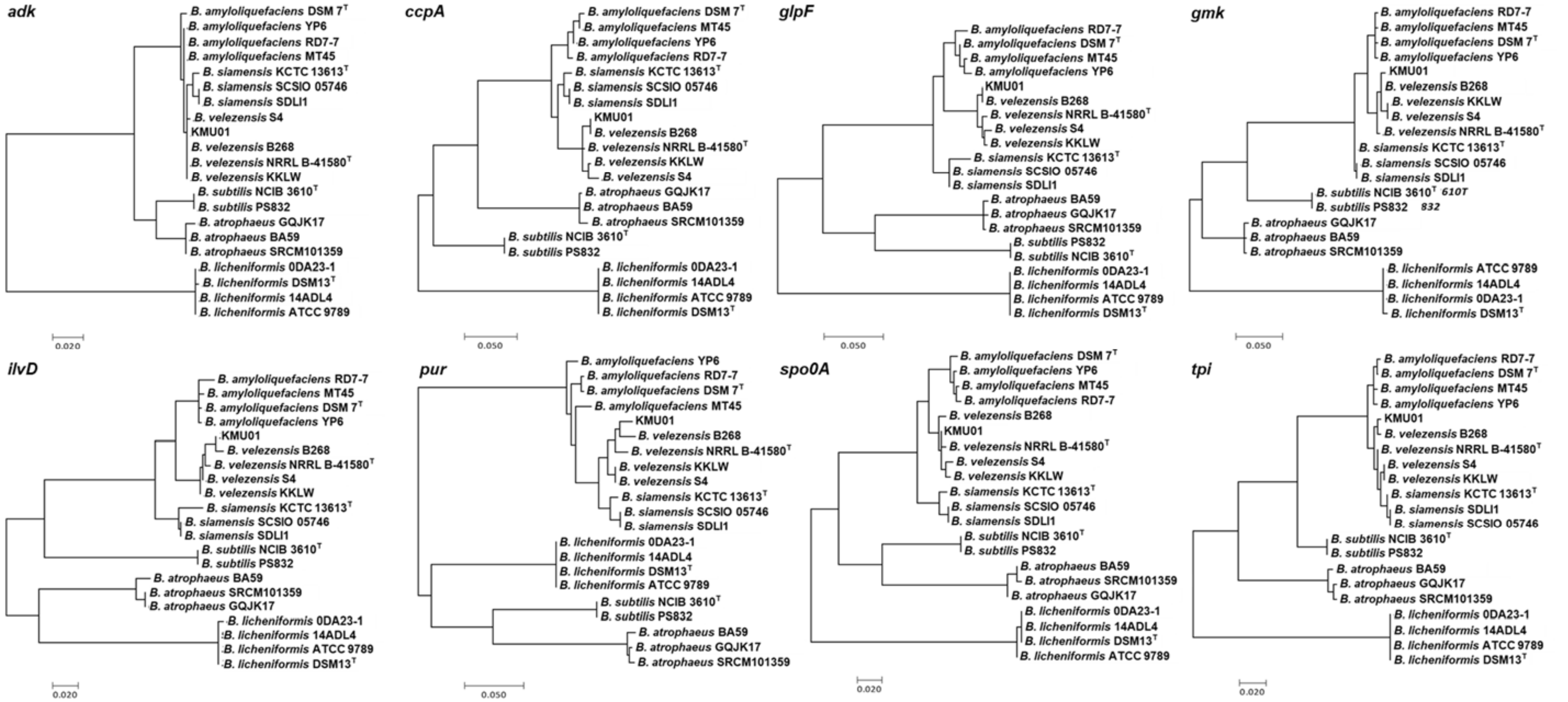

Table S1. Detection of enterotoxin genes in strain KMU01.

Gene	Amplicon Size (bp)	*B. cereus* KCCM 11341	KMU01
nblA	1,154	○	-
nblC	740	○	-
nblD	829	○	-
nheA	499	○	-
nheB	769	○	-
nheC	581	○	-
bceT	924		-

Table S2. List of the genes for salt resistance.

Gene locus	Gene	E.C. No.	Product	COG
Glycine betaine, Proline betaine				
IM712_RS02820	*opuAC*	-	Glycine betaine-binding protein OpuAC	E
IM712_RS02825	*opuAB*	-	Glycine betaine transport system permease protein OpuAB	E
IM712_RS02830	*opuAA*	3.6.3.32	Quaternary-amine-transporting ATPase	E
IM712_RS09990	*opuD*	3.6.3.32	Glycine betaine transporter OpuD	P
Glycerol				
IM712_RS18985	*glpA*	1.1.5.3	Glycerol-3-phosphate dehydrogenase	C
IM712_RS18990	*glpK*	2.7.1.30	Glycerol kinase	C
IM712_RS18995		-	Glycerol uptake facilitator protein	G
IM712_RS19000		-	Glycerol uptake operon antiterminator regulatory protein	K
Cardiolipin				
IM712_RS04970	*cls*	2.7.8.-	hypothetical protein	S
IM712_RS06345	*clsA*	2.7.8.-	Probable cardiolipin synthase YwiE	I
IM712_RS06365	*clsB*	2.7.8.-	Minor cardiolipin synthase ClsB	I
IM712_RS06660	*clsA*	2.7.8.-	Major cardiolipin synthase ClsA	I
IM712_RS08885	*pgpA*	3.1.3.27	Phosphatidylglycerophosphatase	I
IM712_RS13175	*gpsA*	1.1.1.94	Glycerol-3-phosphate dehydrogenase (NAD(P)(+))	C
IM712_RS13720	*plsC*	2.3.1.51	1-acylglycerol-3-phosphate O-acyltransferase	I
IM712_RS14725	*plsY*	2.3.1.15	Glycerol-3-phosphate 1-O-acyltransferase	S
IM712_RS15295	*pgsA*	2.7.8.5	CDP-diacylglycerol--glycerol-3-phosphate 3-phosphatidyltransferase	I
IM712_RS15485	*cdsA*	2.7.7.41	Phosphatidate cytidylyltransferase	I
IM712_RS15815	*plsX*	2.3.1.15	Glycerol-3-phosphate 1-O-acyltransferase	I
IM712_RS18880	*plsC*	2.3.1.51	1-acylglycerol-3-phosphate O-acyltransferase	I
Glucose				
IM712_RS06395	*fbaA*	4.1.2.13	Fructose-bisphosphate aldolase	G
IM712_RS07940	*gapA*	1.2.1.12	Glyceraldehyde-3-phosphate dehydrogenase	G
IM712_RS07950	*tpiA*	5.3.1.1	Triose-phosphate isomerase	G
IM712_RS07955	*gpmB*	5.4.2.12	Phosphoglycerate mutase (2,3-diphosphoglycerate-independent)	E
IM712_RS07960	*eno*	4.2.1.11	Phosphopyruvate hydratase	G
IM712_RS09375	*pgi*	5.3.1.9	Glucose-6-phosphate isomerase	G
IM712_RS10455	*pkfA*	2.7.1.11	6-phosphofructokinase	G
IM712_RS10460	*pyk*	2.7.1.40	Pyruvate kinase	G
IM712_RS10505	*gltA*	2.3.3.1	Citrate (Si)-synthase	C
IM712_RS10565	*gapA*	1.2.1.12	Glyceraldehyde-3-phosphate dehydrogenase	G
IM712_RS12065	*glk*	2.7.1.2	Glucokinase	G
IM712_RS12445	*gltA*	2.3.3.1	Citrate (Si)-synthase	C
IM712_RS13480	*crr*	2.7.1.69	PTS system, glucose-specific IIA component	G
IM712_RS14240	*pgk*	3.4.17.13	Muramoyltetrapeptide carboxypeptidase	V

Gene locus	Gene	E.C. No.	Product	COG
IM712_RS14425	*pgi*	5.3.1.9	Glucose-6-phosphate isomerase	S
IM712_RS15325	*gpmI*	3.4.24.-	Probable inactive metalloprotease YmfF	O
IM712_RS16325	*pyc*	6.4.1.1	Pyruvate carboxylase	C
IM712_RS16605	*pkfB*	2.7.1.11	6-phosphofructokinase	G
IM712_RS18915	*gltA*	2.3.3.1	Citrate (Si)-synthase	C
Glutamate				
IM712_RS06045	*gudB*	1.4.1.2	Glutamate dehydrogenase	E
IM712_RS10510	*icd*	1.1.1.42	Isocitrate dehydrogenase (NADP(+))	C
IM712_RS13110	*gudB*	1.4.1.2	Glutamate dehydrogenase	E
IM712_RS14380	*gltB*	1.4.1.13, 1.4.1.14	Glutamate synthase (NADPH)	E
IM712_RS14385	*gltD*	1.4.1.13, 1.4.1.14	Glutamate synthase (NADPH)	E
IM712_RS14765	*acnA*	4.2.1.3	Aconitate hydratase	C
Proline				
IM712_RS02715		1.2.1.88	L-glutamate gamma-semialdehyde dehydrogenase	C
IM712_RS06050		1.2.1.88	L-glutamate gamma-semialdehyde dehydrogenase	C
IM712_RS12695	*proC*	1.5.1.2	Pyrroline-5-carboxylate reductase	E
IM712_RS14365	*proC*	1.5.1.2	Pyrroline-5-carboxylate reductase	E
IM712_RS14370	*proB*	2.7.2.11	Glutamate 5-kinase	E
IM712_RS17185	*proA*	1.2.1.41	Glutamate-5-semialdehyde dehydrogenase	E
IM712_RS17190	*proB*	2.7.2.11	Glutamate 5-kinase	E
Trehalose				
IM712_RS00595		-	Trehalose operon transcriptional repressor	K
IM712_RS00600	*treC*	3.2.1.93	Trehalose-6-phosphate hydrolase	G
IM712_RS00605	*treB*	2.7.1.69	PTS system, trehalose-specific IIB component	G

The Enzyme Commission (EC) number is a numerical classification scheme for enzymes, based on the chemical reactions they catalyze. The Clusters of Orthologus Group (COG) categorization was generated by annotated gene functions.

Table S3. List of the genes for probiotic properties.

Gene locus	E.C.	Product	Gene	COG
Survivability				
IM712_RS05600	3.5.1.24	Bile salt hydrolase	-	M
Adhesion				
IM712_RS15940	-	Fibronectin-binding protein	-	K
Biofilm formation				
IM712_RS07735	3.1.1.-	Probable secreted lipase	*pnbA*	I
IM712_RS07740	-	HTH-type transcriptional regulator SlrR	*slrR*	K
IM712_RS07745	-	uncharacterized protein	*epsA*	M
IM712_RS07750	2.7.10.2	Non-specific protein-tyrosine kinase	*epsB*	D
IM712_RS07755	-	Probable polysaccharide biosynthesis protein EpsC	*epsC*	M
IM712_RS07760	2.4.-.-	Putative glycosyltransferase EpsD	*epsD*	M
IM712_RS07765	2.4.-.-	Putative glycosyltransferase EpsE	*epsE*	M
IM712_RS07770	2.4.-.-	Putative glycosyltransferase EpsF	*epsF*	M
IM712_RS07775	-	Transmembrane protein EpsG	*epsG*	S
IM712_RS07780	2.4.-.-	Putative glycosyltransferase EpsH	*epsH*	M
IM712_RS07785	2.-.-.-	Putative pyruvyl transferase EpsI	*epsI*	M
IM712_RS07790	2.4.-.-	Chondroitin synthase	*epsJ*	M
IM712_RS07795	-	Uncharacterized membrane protein EpsK	*epsK*	M
IM712_RS07800	2.7.8.36	Undecaprenyl phosphate N,N'-diacetylbacillosamine 1-phosphatetransferase	*pglC*	M
IM712_RS07805	2.3.1.-	Putative acetyltransferase EpsM	*epsM*	S
IM712_RS07810	2.6.1.-	Putative pyridoxal phosphate-dependent aminotransferase EpsN	*epsN*	E
IM712_RS07815	2.-.-.-	Putative pyruvyl transferase EpsO	*epsO*	M
Flagellum				
IM712_RS15515	-	Swarming motility protein SwrB	*swrB*	S
IM712_RS15520	-	RNA polymerase sigma-D factor	*fliA*	K
IM712_RS15525	3.5.1.44	Protein-glutamine glutaminase	*cheD*	NT
IM712_RS15530	-	CheY-P phosphatase CheC	*cheC*	N
IM712_RS15535	-	Chemotaxis protein CheW	*cheW*	NT
IM712_RS15540	2.7.13.3	Histidine kinase	*cheA*	T
IM712_RS15545	-	Chemotaxis response regulator protein-glutamate methylesterase of group 1 operon	*cheB*	T
IM712_RS15550	-	Flagellum site-determining protein YlxH	*ylxH*	D
IM712_RS15555	-	Flagellar biosynthesis protein FlhF	*flhF*	N
IM712_RS15560	-	Flagellar biosynthesis protein FlhA	*flhA*	N
IM712_RS15565	-	Flagellar biosynthetic protein FlhB	*flhB*	N
IM712_RS15570	-	Flagellar biosynthetic protein FliR	*fliR*	N
IM712_RS15575	-	Flagellar biosynthetic protein FliQ	*fliQ*	N
IM712_RS15580	-	Flagellar biosynthetic protein FliP	*fliP*	N
IM712_RS15585	-	Flagellar biosynthetic protein FliZ	*fliZ*	N
IM712_RS15590	-	Chemotaxis protein CheY	*cheY*	T
IM712_RS15595	-	Flagellar motor switch phosphatase FliY	*fliY*	N|N
IM712_RS15600	-	Flagellar motor switch protein FliM	*fliM*	N

Gene locus	E.C.	Product	Gene	COG
IM712_RS15605	-	Flagellar protein FliL	*fliL*	N
IM712_RS15610	-	uncharacterized protein	*flbD*	N
IM712_RS15615	-	Flagellar hook protein FlgE	*flgE*	N
IM712_RS15620	-	FlaA locus uncharacterized protein YlxG	*ylxG*	N
IM712_RS15625	-	Probable flagellar hook-length control protein	*fliK*	N
IM712_RS15630	-	FlaA locus 22.9 kDa protein	*flaA*	S
IM712_RS15635	-	Flagellar FliJ protein	*fliJ*	N
IM712_RS15640	3.6.3.14	H(+)-transporting two-sector ATPase	*fliI*	NU
IM712_RS15645	-	Probable flagellar assembly protein FliH	*fliH*	N
IM712_RS15650	-	Flagellar motor switch protein FliG	*fliG*	N
IM712_RS15655	-	Flagellar M-ring protein	*fliF*	N
IM712_RS15660	-	Flagellar hook-basal body complex protein FliE	*fliE*	N
IM712_RS15665	-	Flagellar basal-body rod protein FlgC	*fliC*	N
IM712_RS15670	-	Flagellar basal body rod protein FlgB	*fliB*	N
Bacteriocin				
IM712_RS05185	-	Peptidase domain-containing ABC transporter/ bacteriocin export protein		V
IM712_RS05190	-	Lacticin 481/lactococcin biosynthesis protein lcnDR2	*lcnDR2*	V
IM712_RS05195	4.1.1.36, 6.3.2.5	Phosphopantothenoylcysteine decarboxylase/ Modification protein		H
IM712_RS05200	-	Hypothetical protein		T
IM712_RS05205	-	Lantibiotic mersacidin		
Spore Cortex				
IM712_RS12890	-	Stage V sporulation protein AA	*spoVAA*	S
IM712_RS12895	-	Stage V sporulation protein	*spoVAB*	S
IM712_RS12900	-	Stage V sporulation protein AC	*spoVAC*	S
IM712_RS12905	-	Stage V sporulation protein AD	*spoVAD*	S
IM712_RS12910	-	Stage V sporulation protein AEB	*spoVAEb*	S
IM712_RS12915	-	Stage V sporulation protein AE	*spoVAEa*	S
IM712_RS12920	-	Stage V sporulation protein AF	*spoVAF*	S
Spore coat				
IM712_RS01655	-	Spore coat protein F-like protein YraG	*yraG*	S
IM712_RS01660	-	Spore coat protein F-like protein YraF	*yraF*	M
IM712_RS01665	1.1.1.284, 1.1.1.1	S-(hydroxymethyl)glutathione dehydrogenase		E
IM712_RS01670	-	uncharacterized protein		S
IM712_RS01675	-	Spore coat protein F-like protein YraD	*yraD*	M
IM712_RS17835	-	Spore coat protein	*cotV*	S
IM712_RS17840	-	Spore coat protein	*cotW*	
IM712_RS17845	-	Spore coat protein X	*cotX*	S
IM712_RS17850	-	Spore coat protein Y	*cotY*	
IM712_RS17855	-	Spore coat protein	*cotZ*	
IM712_RS17860	-	Spore coat protein		M
Spore Wall				
IM712_RS05985	2.4.1.293	GalNAc(5)-diNAcBac-PP-undecaprenol beta-1,3-glucosyltransferase	*spsA*	M

Gene locus	E.C.	Product	Gene	COG
IM712_RS05990	-	Spore coat polysaccharide biosynthesis protein SpsB	*spsB*	M
IM712_RS05995	2.6.1.87	UDP-4-amino-4-deoxy-L-arabinose aminotransferase	*spsC*	E
IM712_RS06000	2.3.1.210	dTDP-4-amino-4,6-dideoxy-D-galactose acyltransferase	*spsD*	S
IM712_RS06005	2.5.1.56	N-acetylneuraminate synthase	*spsE*	M
IM712_RS06010	-	Spore coat polysaccharide biosynthesis protein SpsF	*spsF*	M
IM712_RS06015	-	Spore coat polysaccharide biosynthesis protein SpsG	*spsG*	M
IM712_RS06020	2.7.7.24	Glucose-1-phosphate thymidylyltransferase	*spsI*	M
IM712_RS06025	4.2.1.46	dTDP-glucose 4,6-dehydratase	*spsJ*	M
IM712_RS06030	1.1.1.133	dTDP-4-dehydrorhamnose reductase	*spsK*	M
IM712_RS06035	5.1.3.13	dTDP-4-dehydrorhamnose 3,5-epimerase	*spsL*	M
Spore germination				
IM712_RS07060	-	Spore germination protein B3	*gerAC*	S
IM712_RS07065	-	Spore germination protein B2	*gerKB*	S
IM712_RS07070	-	Spore germination protein	*gerKA*	S
IM712_RS18310	-	Probable spore germination protein GerPA	*gerPA*	S
IM712_RS18315	-	Probable spore germination protein GerPB	*gerPB*	S
IM712_RS18320	-	Probable spore germination protein GerPC	*gerPC*	S
IM712_RS18325	-	Probable spore germination protein GerPD	*gerPD*	S
IM712_RS18330	-	Probable spore germination protein GerPE	*gerPE*	S
IM712_RS18335	-	Probable spore germination protein GerPF	*gerPF*	S
Others				
IM712_RS00225	-	Small, acid-soluble spore protein gamma-type		S
IM712_RS00425	-	Small, acid-soluble spore protein		M
IM712_RS00620	-	Spore coat protein		O
IM712_RS02295	-	Spore germination lipase LipC		E
IM712_RS02525	-	Spore germination protein KB		S
IM712_RS02530	-	Spore germination protein KC		S
IM712_RS02535	-	Spore germination protein KA		S
IM712_RS03525	-	Spore germination protein GerD	*gerD*	S
IM712_RS04085	-	Spore protein YabQ		S
IM712_RS04090	-	Spore protein YabP		S
IM712_RS04340	-	Spore germination protein YaaH	*yaaH*	M
IM712_RS05880	-	Spore morphoproteinis and germination protein YwcE		-
IM712_RS05980	-	Spore coat protein GerQ	*gerQ*	S
IM712_RS06175	-	Prespore-specific transcriptional regulator RsfA		K
IM712_RS06915	-	Spore coat protein		S
IM712_RS06920	-	Inner spore coat protein		M
IM712_RS06925	-	Spore coat protein		S
IM712_RS06995	-	Putative spore germination protein YfkR	*yfkR*	S
IM712_RS07000	-	Spore germination protein B2		S
IM712_RS08350	-	Small, acid-soluble spore protein J		-
IM712_RS08485	-	Spore germination protein A3		S
IM712_RS08490	-	Spore germination protein A2		S
IM712_RS08495	-	Spore germination protein A1		S

Gene locus	E.C.	Product	Gene	COG
IM712_RS08860	-	L-Ala--D-Glu endopeptidase		M
IM712_RS08890	-	Endospore coat-associated protein YutH		S
IM712_RS09150	-	Spore germination protein-like protein YueG	*yueG*	S
IM712_RS10360	-	Uncharacterized spore protein YtfJ		S
IM712_RS10730	-	Small, acid-soluble spore protein		M
IM712_RS10855	-	Spore germination protein GerE	*gerE*	K
IM712_RS10870	-	Spore germination protein GerM	*gerM*	S
IM712_RS11040	-	Stage II sporulation protein		
IM712_RS11150	-	Spore coat assembly protein ExsA		S
IM712_RS12180	-	Spore coat-associated protein		S
IM712_RS12385	-	Spore germination protein-like protein YpzD		
IM712_RS12390	-	Spore germination protein-like protein YpzD		
IM712_RS13010	-	Spore maturation protein		S
IM712_RS13015	-	Spore maturation protein		S
IM712_RS13450	-	Small, acid-soluble spore protein		
IM712_RS13500	-	Spore coat protein		S
IM712_RS13620	-	Small, acid-soluble spore protein		
IM712_RS13665	-	Spore coat protein		M
IM712_RS14065	-	Spore germination protein GerT	*gerT*	O
IM712_RS14745	-	Small, acid-soluble spore protein Tlp		M
IM712_RS14750	-	Small, acid-soluble spore protein		
IM712_RS14770	-	Small, acid-soluble spore protein		M
IM712_RS14775	-	Small, acid-soluble spore protein		M
IM712_RS14780	-	Spore coat protein		O
IM712_RS15240	-	Spore coat protein		M
IM712_RS16815	4.1.99.14	Spore photoproduct lyase	*splB*	L
IM712_RS17035	-	Small, acid-soluble spore protein		S
IM712_RS18765	-	Endospore coat-associated protein YheC		S
IM712_RS18770	-	Endospore coat-associated protein YheD		S
IM712_RS18780	-	Small, acid-soluble spore protein		S
IM712_RS19045	-	Spore coat protein F-like protein YhcQ	*yhcQ*	M
Health enhancement				
IM712_RS06900	2.3.2.2	Gamma-glutamyltransferase	*ggt*	E
IM712_RS09030	3.4.11.1	Leucyl aminopeptidase	*pepA*	E
IM712_RS14420	2.3.2.2	Gamma-glutamyltransferase	*ggt*	E
IM712_RS18500	3.4.21.62	Nattokinase	*aprE*	O
IM712_RS16425	4.1.1.19	Arginine decarboxylase	*speA*	E
IM712_RS06235	3.5.3.11	Agmatinase	*speB*	E
IM712_RS15005	6.3.1.11	Gamma-glutamyl-putrescine synthetase	*puuA*	E
IM712_RS18465	1.4.3.-	Gamma-glutamyl-putrescine oxidase	*puuB*	CE
IM712_RS00790	1.2.1.-	Gamma-glutamyl-aminobutyraldehyde dehydrogenase	*puuC*	C
IM712_RS05085	1.2.1.-	Gamma-glutamyl-aminobutyraldehyde dehydrogenase	*puuC*	C
IM712_RS10275	1.2.1.-	Gamma-glutamyl-aminobutyraldehyde dehydrogenase	*puuC*	C
IM712_RS14165	1.2.1.-	Gamma-glutamyl-aminobutyraldehyde dehydrogenase	*puuC*	C

Gene locus	E.C.	Product	Gene	COG
IM712_RS01635	3.5.1.94	Gamma-glutamyl-gamma-aminobutyrate hydrolase family protein	*puuD*	S

The Enzyme Commission (EC) number is a numerical classification scheme for enzymes, based on the chemical reactions they catalyze. The Clusters of Orthologus Group (COG) categorization was generated by annotated gene functions.

Table S4. List of the genes for amino acid synthesis.

Gene locus	E.C. No.	Product	Gene	COG
IM712_RS00555	4.2.1.10	3-dehydroquinate dehydratase	*aroD*	E
IM712_RS00560	1.1.1.25	Shikimate dehydrogenase	*aroE*	E
IM712_RS00675	1.14.13.165	Nitric-oxide synthase (NAD(P)H)	*nos*	EP
IM712_RS01120	6.3.4.13	Phosphoribosylamine--glycine ligase	*purD*	F
IM712_RS01125	2.1.2.3, 3.5.4.10	Phosphoribosylaminoimidazolecarboxamide formyltransferase	*purH*	F
IM712_RS01130	2.1.2.2	Phosphoribosylglycinamide formyltransferase	*purN*	F
IM712_RS01135	6.3.3.1	Phosphoribosylformylglycinamidine cyclo-ligase	*purM*	F
IM712_RS01140	2.4.2.14	Amidophosphoribosyltransferase	*purF*	F
IM712_RS01145	6.3.5.3	Phosphoribosylformylglycinamidine synthase	*purL*	F
IM712_RS01150	6.3.5.3	Phosphoribosylformylglycinamidine synthase	*purL*	F
IM712_RS01155	6.3.5.3	Phosphoribosylformylglycinamidine synthase	*purL*	F
IM712_RS01160	6.3.2.6	Phosphoribosylaminoimidazolesuccinocarboxamide synthase	*purC*	F
IM712_RS01165	4.3.2.2	Adenylosuccinate lyase	*purB*	F
IM712_RS01170	6.3.4.18	5-(carboxyamino)imidazole ribonucleotide synthase	*purK*	F
IM712_RS01175	5.4.99.18	5-(carboxyamino)imidazole ribonucleotide mutase	*purE*	F
IM712_RS01220	6.3.5.2	GMP synthase (glutamine-hydrolyzing)	*guaA*	F
IM712_RS01750	4.3.3.7	4-hydroxy-tetrahydrodipicolinate synthase	*dapA*	E
IM712_RS01999	6.3.5.4	Asparagine synthase (glutamine-hydrolyzing)	*asnB*	E
IM712_RS02390	1.2.1.16, 1.2.1.79, 1.2.1.20	Succinate-semialdehyde dehydrogenase (NAD(P)(+))	*gabD*	C
IM712_RS02395	2.6.1.19, 2.6.1.22	4-aminobutyrate--2-oxoglutarate transaminase	*gabT*	E
IM712_RS02450	2.7.2.4	Aspartate kinase	*lysC*	E
IM712_RS02715	1.2.1.88	L-glutamate gamma-semialdehyde dehydrogenase		C
IM712_RS02735	2.7.1.71	Shikimate kinase	*aroK*|*aroL*	E
IM712_RS03035	3.5.1.2	Glutaminase	*glsA*	E
IM712_RS03045	2.1.1.10	Homocysteine S-methyltransferase	*mmuM*	E
IM712_RS03055	2.6.1.42	Branched-chain-amino-acid transaminase	*ilvE*	E
IM712_RS03090	2.1.2.2	Phosphoribosylglycinamide formyltransferase	*purT*	F
IM712_RS03375	2.5.1.47	Cysteine synthase	*cysK*	K
IM712_RS03707	6.3.5.4	Asparagine synthase (glutamine-hydrolyzing)	*asnB*	E
IM712_RS03840	2.3.1.30	Serine O-acetyltransferase	*cysE*	E
IM712_RS04000	2.6.1.42	Branched-chain-amino-acid transaminase	*ilvE*	E
IM712_RS04015	2.5.1.47	Cysteine synthase	*cysK*	E
IM712_RS04020	5.2.1.8	Peptidylprolyl isomerase	*prsA*	O
IM712_RS04135	2.7.6.1	Ribose-phosphate diphosphokinase	*prsA*	F
IM712_RS04385	1.1.1.205	IMP dehydrogenase	*guaB*	FS
IM712_RS04795	6.3.4.4	Adenylosuccinate synthase	*purA*	F
IM712_RS04900	2.6.1.13	Ornithine aminotransferase	*rocD*	E
IM712_RS04910	3.5.3.1	Arginase	*rocF*	E
IM712_RS05320	3.5.3.8	Formimidoylglutamase	*hutG*	E
IM712_RS05325	3.5.2.7	Imidazolonepropionase	*hutI*	Q

Gene locus	E.C. No.	Product	Gene	COG
IM712_RS05330	4.2.1.49	Urocanate hydratase	*hutU*	E
IM712_RS05335	4.3.1.3	Histidine ammonia-lyase	*hutH*	E
IM712_RS05515	2.1.1.14	5-methyltetrahydropteroyltriglutamate--homocysteine S-methyltransferase	*metE*	E
IM712_RS05700	2.6.1.42	Branched-chain-amino-acid transaminase	*ilvE*	E
IM712_RS06050	1.2.1.88	L-glutamate gamma-semialdehyde dehydrogenase		C
IM712_RS06395	4.1.2.13	Fructose-bisphosphate aldolase	*fbaA*	G
IM712_RS06505	2.1.2.1	Glycine hydroxymethyltransferase	*glyA*	E
IM712_RS06945	2.2.1.6	Acetolactate synthase	*ilvB|ilvG|ilvI*	E
IM712_RS07470	-	ATP phosphoribosyltransferase regulatory subunit	*hisZ*	E
IM712_RS07475	2.4.2.17	ATP phosphoribosyltransferase	*hisG*	E
IM712_RS07480	1.1.1.23	Histidinol dehydrogenase	*hisD*	E
IM712_RS07485	4.2.1.19	Imidazoleglycerol-phosphate dehydratase	*hisB*	E
IM712_RS07490	2.4.2.-	Imidazole glycerol phosphate synthase subunit HisH	*hisH*	E
IM712_RS07495	5.3.1.16	1-(5-phosphoribosyl)-5-((5-phosphoribosylamino)methylideneamino)imidazole-4-carboxamideisomerase	*hisA*	E
IM712_RS07500	4.1.3.-	Imidazole glycerol phosphate synthase subunit HisF	*hisF*	E
IM712_RS07505	3.6.1.31, 3.5.4.19	Phosphoribosyl-ATP diphosphatase	*hisIE*	E
IM712_RS07940	1.2.1.12	Glyceraldehyde-3-phosphate dehydrogenase (phosphorylating)	*gapA*	G
IM712_RS07945	2.7.2.3	Phosphoglycerate kinase	*pgk*	G
IM712_RS07955	5.4.2.12	Phosphoglycerate mutase (2,3-diphosphoglycerate-independent)	*gpmI*	G
IM712_RS07960	4.2.1.11	Phosphopyruvate hydratase	*eno*	G
IM712_RS08505	4.2.1.2	Fumarate hydratase	*fumC*	C
IM712_RS08605	1.5.-.-	Proline dehydrogenase	*putB*	E
IM712_RS08895	1.1.1.3	Homoserine dehydrogenase	*hom*	E
IM712_RS08900	4.2.3.1	Threonine synthase	*thrC*	E
IM712_RS08905	2.7.1.39	Homoserine kinase	*thrB1*	E
IM712_RS08940	5.1.1.7	Diaminopimelate epimerase	*dapF*	E
IM712_RS09375	5.3.1.9	Glucose-6-phosphate isomerase	*pgi*	G
IM712_RS09825	2.5.1.47	Cysteine synthase	*cysK*	E
IM712_RS10135	5.4.99.5, 4.2.1.51	Chorismate mutase	*pheA*	E
IM712_RS10385	6.3.4.5	Argininosuccinate synthase	*argG*	E
IM712_RS10390	4.3.2.1	Argininosuccinate lyase	*argH*	E
IM712_RS10405	1.1.1.205	IMP dehydrogenase	*guaB*	K
IM712_RS10455	2.7.1.11	6-phosphofructokinase	*pfkA*	G
IM712_RS10460	2.7.1.40	Pyruvate kinase	*pyk*	G
IM712_RS10515	1.1.1.37	Malate dehydrogenase	*mdh*	C
IM712_RS10565	1.2.1.12	Glyceraldehyde-3-phosphate dehydrogenase (phosphorylating)	*gapA*	G
IM712_RS10825	2.7.2.4	Aspartate kinase	*lysC*	E
IM712_RS10840	1.3.5.1, 1.3.5.4	Succinate dehydrogenase (quinone)	*sdhA|frdA*	C
IM712_RS10910	2.2.1.6	Acetolactate synthase large subunit	*IlvB*	E
IM712_RS10915	2.2.1.6	acetolactate synthase small subunit	*ilvN*	E

Gene locus	E.C. No.	Product	Gene	COG
IM712_RS10920	1.1.1.86	Ketol-acid reductoisomerase (NADP(+))	*ilvC*	E
IM712_RS10930	1.1.1.85	3-isopropylmalate dehydrogenase	*leuB*	E
IM712_RS10935	4.2.1.33, 4.2.1.35	3-isopropylmalate dehydratase	*leuC*	E
IM712_RS10940	4.2.1.33, 4.2.1.35	3-isopropylmalate dehydratase	*leuD*	E
IM712_RS11115	5.4.99.5	Chorismate mutase	*pheB*	E
IM712_RS11120	5.4.99.5, 4.2.1.51	Chorismate mutase	*pheA*	E
IM712_RS11425	2.5.1.47	Cysteine synthase	*cysK*	E
IM712_RS11665	1.1.1.25	Shikimate dehydrogenase	*aroE*	E
IM712_RS12695	1.5.1.2	Pyrroline-5-carboxylate reductase	*proC*	E
IM712_RS12720	5.4.2.12	Phosphoglycerate mutase (2,3-diphosphoglycerate-independent)	*gpmB*	E
IM712_RS12925	4.1.1.20	Diaminopimelate decarboxylase	*lysA*	E
IM712_RS13235	4.2.3.5	Chorismate synthase	*aroC*	E
IM712_RS13240	4.2.3.4	3-dehydroquinate synthase	*aroB*	E
IM712_RS13250	4.1.3.27	Anthranilate synthase	*trpE*	E
IM712_RS13255	2.4.2.18	Anthranilate phosphoribosyltransferase	*trpD*	E
IM712_RS13260	4.1.1.48	Indole-3-glycerol-phosphate synthase	*trpC*	E
IM712_RS13265	5.3.1.24	Phosphoribosylanthranilate isomerase	*trpF*	E
IM712_RS13270	4.2.1.20	Tryptophan synthase	*trpB*	E
IM712_RS13275	4.2.1.20	Tryptophan synthase	*trpA*	E
IM712_RS13280	2.6.1.9	Histidinol-phosphate transaminase	*hisC*	E
IM712_RS13290	2.5.1.19	3-phosphoshikimate 1-carboxyvinyltransferase	*aroA*	E
IM712_RS13350	1.17.1.8	4-hydroxy-tetrahydrodipicolinate reductase	*dapB*	E
IM712_RS13410	2.6.1.1	Aspartate transaminase	*aspB*	E
IM712_RS13480	2.7.1.69	Protein-N(pi)-phosphohistidine--sugar phosphotransferase	*crr*	G
IM712_RS13670	2.3.1.46	Homoserine O-succinyltransferase	*metA*	E
IM712_RS13690	4.2.1.9	Dihydroxy-acid dehydratase	*ilvD*	E
IM712_RS13735	4.3.1.19	Threonine ammonia-lyase	*ilvA*|*tdcB*	E
IM712_RS14365	1.5.1.2	Pyrroline-5-carboxylate reductase	*proC*	E
IM712_RS14370	2.7.2.11	Glutamate 5-kinase	*proB*	E
IM712_RS14380	1.4.1.13, 1.4.1.14	Glutamate synthase (NADPH)	*gltB*	E
IM712_RS14385	1.4.1.13, 1.4.1.14	Glutamate synthase (NADPH)	*gltD*	E
IM712_RS14425	5.3.1.9	Glucose-6-phosphate isomerase	*pgi*	S
IM712_RS14820	2.2.1.1	Transketolase	*tktA*|*tktB*	G
IM712_RS15005	6.3.1.2	Glutamate--ammonia ligase	*glnA*	E
IM712_RS15370	4.3.3.7	4-hydroxy-tetrahydrodipicolinate synthase	*dapA*	E
IM712_RS15375	2.7.2.4	Aspartate kinase	*lysC*	E
IM712_RS15380	1.2.1.11	Aspartate-semialdehyde dehydrogenase	*asd*	E
IM712_RS16325	6.4.1.1	Pyruvate carboxylase	*pyc*	C
IM712_RS16340	3.5.1.2	Glutaminase	*glsA*	E
IM712_RS16405	3.1.3.25, 3.1.3.93, 3.1.3.15	Inositol-phosphate phosphatase	IMPL2	S
IM712_RS16605	2.7.1.11	6-phosphofructokinase	*pfkB*	G

Gene locus	E.C. No.	Product	Gene	COG
IM712_RS16690	3.5.1.47	N-acetyldiaminopimelate deacetylase	*dapL*	E
IM712_RS16695	2.3.1.89	Tetrahydrodipicolinate N-acetyltransferase	*dapH|dapD*	E
IM712_RS16785	2.6.1.-	Putative N-acetyl-LL-diaminopimelate aminotransferase	*patA*	E
IM712_RS17135	2.1.1.14	5-methyltetrahydropteroyltriglutamate--homocysteine S-methyltransferase	*metE*	E
IM712_RS17185	1.2.1.41	Glutamate-5-semialdehyde dehydrogenase	*proA*	E
IM712_RS17190	2.7.2.11	Glutamate 5-kinase	*proB*	E
IM712_RS17765	2.5.1.48	Cystathionine gamma-synthase	*metB*	E
IM712_RS18110	2.1.3.3	Ornithine carbamoyltransferase	*argF|argI*	E
IM712_RS18225	2.1.1.10	Homocysteine S-methyltransferase	*mmuM*	E
IM712_RS18280	6.3.5.4	Asparagine synthase (glutamine-hydrolyzing)	*asnB*	E
IM712_RS18675	5.2.1.8	Peptidylprolyl isomerase	*prsA*	O
IM712_RS18815	2.6.1.21	D-amino-acid transaminase	*dat*	E
IM712_RS18865	2.6.1.1	Aspartate transaminase	*yhdR*	E
IM712_RS19020	1.1.1.205	IMP dehydrogenase	*guaB*	S

Table S5. Potential enzyme genes identified in KMU01 genome for a starter strain.

Gene locus	E.C. No.	Product	COG
Amylase			
IM712_RS02800	3.2.1.1	Alpha-amylase	G
Lipase			
IM712_RS02935	3.1.1.3	Triacylglycerol lipase	S
IM712_RS07735	3.1.1.-	Probable secreted lipase	I
IM712_RS07745	3.1.1.1	Carboxylesterase	S
IM712_RS09840	3.1.1.5	Lysophospholipase	I
IM712_RS11500	3.1.4.54	N-acetylphosphatidylethanolamine-hydrolyzing phospholipase D	S
Protease			
IM712_RS00285	3.4.21.19	Glutamyl endopeptidase	O
IM712_RS00705	3.4.21.96	Lactocepin	O
IM712_RS02020	-	Putative rhomboid protease YdcA	S
IM712_RS02905	3.4.-.-	Peptidoglycan L-alanyl-D-glutamate endopeptidase CwlK	M
IM712_RS04035	3.4.24.-	ATP-dependent zinc metalloprotease FtsH	O
IM712_RS04175	-	Sporulation-specific protease YabG	S
IM712_RS04790	3.4.21.26	Prolyl oligopeptidase	S
IM712_RS04845	3.4.21.107	Peptidase Do	O
IM712_RS05045	3.4.24.-	hypothetical protein	O
IM712_RS05745	3.4.21.-	Minor extracellular protease Epr	O
IM712_RS05905	3.4.21.-	Minor extracellular protease vpr	O
IM712_RS06210	-	Putative zinc metalloprotease YwhC	S
IM712_RS07340	3.4.21.102	C-terminal processing peptidase	M
IM712_RS07630	3.4.21.92	Endopeptidase Clp	O
IM712_RS07965	3.4.21.-	Aminopeptidase YwaD	S
IM712_RS08530	3.4.21.107	Peptidase Do	O
IM712_RS08545	3.4.24.-	Group B oligopeptidase PepB	E
IM712_RS09405	-	Putative membrane protease YugP	S
IM712_RS10345	3.4.21.-	Putative signal peptide peptidase SppA	OU
IM712_RS10960	3.4.21.-	Lon protease	O
IM712_RS10965	3.4.21.53	Endopeptidase La	O
IM712_RS11035	3.4.23.43, 2.1.1.-	Type 4 prepilin-like protein leader peptide-processing enzyme	NOU
IM712_RS11085	3.4.24.-	Stage IV sporulation protein FB	S
IM712_RS11380	3.4.-.-	Uncharacterized protease YrrN	O
IM712_RS11385	3.4.-.-	Uncharacterized protease YrrO	O
IM712_RS11735	3.4.24.78	GPR endopeptidase	O
IM712_RS12175	3.4.-.-	Signal peptidase I	U
IM712_RS12370	3.4.21.116	SpoIVB peptidase	M
IM712_RS12945	3.4.21.89	Signal peptidase I	U
IM712_RS13120	-	Protease PrsW	S

Gene locus	E.C. No.	Product	COG
IM712_RS14000	3.4.21.102	C-terminal processing peptidase	M
IM712_RS14110	3.4.-.-	D-gamma-glutamyl-meso-diaminopimelic acid endopeptidase CwlS	M|M
IM712_RS15110	3.4.21.-	Serine protease AprX	O
IM712_RS15320	3.4.24.56	Insulysin	O
IM712_RS15325	3.4.24.-	Probable inactive metalloprotease YmfF	O
IM712_RS15360	3.4.21.92	Endopeptidase Clp	O
IM712_RS15400	3.4.24.-	Uncharacterized zinc protease YmxG	O
IM712_RS15475	3.4.24.-	Probable protease eep	M
IM712_RS15685	3.4.25.2	HslU--HslV peptidase	O
IM712_RS16040	3.4.23.36	Signal peptidase II	MU
IM712_RS16110	3.4.23.-	Sporulation sigma-E factor-processing peptidase	S
IM712_RS16115	3.4.21.-	Bacillopeptidase	O|S
IM712_RS16395	3.4.24.28	Bacillolysin	E
IM712_RS16595	3.4.21.89	Signal peptidase I	U
IM712_RS17025	3.4.24.-	Protease HtpX like protein	O
IM712_RS17130	3.4.21.-	Major intracellular serine protease	O
IM712_RS17235	3.4.-.-	Gamma-D-glutamyl-L-lysine endopeptidase	M
IM712_RS17960	3.4.24.-	Oligoendopeptidase F like protein	E
IM712_RS18400	3.4.21.89	Signal peptidase I	U
IM712_RS18500	3.4.21.62	Subtilisin	O
IM712_RS18505	3.4.24.84	Ste24 endopeptidase	O
IM712_RS18925	3.4.-.-	Probable peptidoglycan endopeptidase LytE	M|M
IM712_RS19035	3.4.22.70	Sortase A	M

The Enzyme Commission (EC) number is a numerical classification scheme for enzymes, based on the chemical reactions they catalyze. The Clusters of Orthologus Group (COG) categorization was generated by annotated gene functions.
